# Phase-to-intensity conversion of magnonic spin currents and application to the design of a majority gate

**DOI:** 10.1038/srep38235

**Published:** 2016-12-01

**Authors:** T. Brächer, F. Heussner, P. Pirro, T. Meyer, T. Fischer, M. Geilen, B. Heinz, B. Lägel, A. A. Serga, B. Hillebrands

**Affiliations:** 1 Univ. Grenoble Alpes, CNRS, CEA, INAC-SPINTEC, 17, rue des Martyrs 38054, Grenoble, France; 2 Graduate School Materials Science in Mainz, Gottlieb-Daimler-Strasse 47, D-67663 Kaiserslautern, Germany; 3 Fachbereich Physik and Forschungszentrum OPTIMAS, Technische Universität Kaiserslautern, D-67663 Kaiserslautern, Germany

## Abstract

Magnonic spin currents in the form of spin waves and their quanta, magnons, are a promising candidate for a new generation of wave-based logic devices beyond CMOS, where information is encoded in the phase of travelling spin-wave packets. The direct readout of this phase on a chip is of vital importance to couple magnonic circuits to conventional CMOS electronics. Here, we present the conversion of the spin-wave phase into a spin-wave intensity by local non-adiabatic parallel pumping in a microstructure. This conversion takes place within the spin-wave system itself and the resulting spin-wave intensity can be conveniently transformed into a DC voltage. We also demonstrate how the phase-to-intensity conversion can be used to extract the majority information from an all-magnonic majority gate. This conversion method promises a convenient readout of the magnon phase in future magnon-based devices.

In the quest for new efficient methods for information processing beyond CMOS, the utilization of spin waves and magnons, their quanta, constitutes a highly promising approach. Consequently, the research field of magnonics^1^, an innovative branch of spintronics focusing on the potential applications of magnonic spin currents, has attracted large research interest in recent years and lead to the formulation of several engineering concepts employing magnons^2^,^3^,^4^. This is motivated by the prospect of miniaturized spin-wave-based logic devices with low power consumption and a high data density due to parallel processing^1^,^5^,^6^,^7^,^8^. The realization of a XNOR logic gate^9^, the design of a spin-wave majority gate^10^,^11^,^12^, the progress towards graded-index magnonics^13^, the development of a magnonic beam splitter^14^ and a spin-wave multiplexer^15^ as well as the experimental realization of a magnon transistor^16^ are only a few instructive examples. They show that spin-wave-based approaches, which utilize the phase of the waves as parameter for data processing, can largely reduce the amount of basic logic elements needed compared to conventional CMOS architecture. To combine magnon-based data processing with CMOS technology, the transformation of the information carried by spin waves directly on the chip into an electric signal, which can be used in subsequent electronic circuits, is of crucial importance. However, state-of-the-art schemes to convert AC spin waves into DC voltages, such as the inverse spin Hall effect or the tunnelling magnetoresistance effect^17^,^18^,^19^,^20^,^21^, are not sensitive to the spin-wave phase. Hence, the electrical readout of the phase information is a challenging, yet crucial, task for the success of a spin-wave-based logic.

In this work, we experimentally demonstrate a method to convert the phase information of travelling spin waves into an amplified spin-wave intensity, which can be readout conveniently by standard methods. This phase-to-intensity conversion is based on phase-dependent spin-wave amplification by means of non-adiabatic parallel pumping^22^,^23^,^24^,^25^,^26^,^27^, a process which takes place within the spin-wave system itself. We have realized this phase-to-intensity conversion for spin waves in a microstructured magnonic waveguide made from Ni_81_Fe_19_ (Permalloy, Py). Thus, this concept is demonstrated at dimensions that meet the requirements for magnon-based applications and can be readily scaled down even further. By using microfocused Brillouin light scattering spectroscopy (BLS)^28^,^29^, we present the continuous variation of the parametric amplification gain of a propagating, coherent, signal-carrying spin wave depending on its phase relation to the pumping field. To illustrate the direct integration of the conversion process into a realistic spin-wave logic device, by means of micromagnetic simulations, we show how the process of non-adiabatic parallel pumping can be applied to a magnon-based majority gate, one of the key elements of magnon-based logics^4^,^30^. The experiment and the simulations demonstrate that parallel pumping constitutes a very powerful and versatile tool to readout the spin-wave phase in microscopic devices. Due to its inherent increase of the spin-wave intensity, it further facilitates spin-wave detection. This increase of the spin-wave intensity only at interconnects between the spin-wave system and its periphery allows to keep the spin-wave intensity in the conduits at a minimum, hence, reducing energy costs and avoiding non-linear spin-wave interactions.

## Non-adiabatic parallel pumping as a tool for the phase-to-intensity conversion

Parallel pumping of spin waves^22^,^23^,^24^,^25^,^26^,^27^ is the application of parametric amplification or parametric resonance, a common phenomenon in many areas of physics^31^,^32^,^33^,^34^,^35^, to the spin-wave manifold in a magnetic material. In contrast to, for instance, optical parametric amplification, where photons are converted into photons, or in contrast to other spin-wave instabilities, where magnons are converted into magnons, parallel pumping constitutes a photon-magnon interaction. Furthermore, it commonly leads to the amplification of spin-waves at one half of the pumping frequency. The photon-magnon interaction originates from the coupling of a time-varying effective field, named pumping field (frequency *f*_p_, effective wave number *k*_p_, phase *ϕ*_p_), to the longitudinal component of an already present dynamic magnetization, e.g., an input signal spin wave with a frequency at half of the pumping frequency. In the present work, the pumping field is provided by a local microwave field. Due to this coupling, pairs of magnons are created by the splitting of microwave photons from the pumping field. One of these magnons contributes to the amplification of the signal spin wave (frequency *f*_s_, wave number *k*_s_, phase *ϕ*_s_) and the other to the formation of an additional wave known as the idler spin wave (frequency *f*_i_, wave number *k*_i_, phase *ϕ*_i_). It should be noted that, in general, the magnon wave vector is much larger than the photon wave vector. The amplification gain of the signal wave is significant if the dissipation losses are compensated by the parametrically injected magnons, which is described by the parametric instability threshold^24^.

During the process of parallel pumping, energy and momentum are conserved^23^


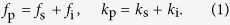


Moreover, for an efficient operation of the parametric amplifier, the phases of the created magnons have to satisfy^24^,^25^


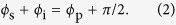


The phase of the signal spin waves *ϕ*_s_ and the phase of the pumping field *ϕ*_p_ can be controlled externally. Consequently, the phase of the idler wave is selected by the spin-wave system in order to satisfy Eq. 2. In the following, we will discuss how this general relation can be used to transform the phase *ϕ*_s_, into which information is encoded, into intensity information.

**Figure 1:**
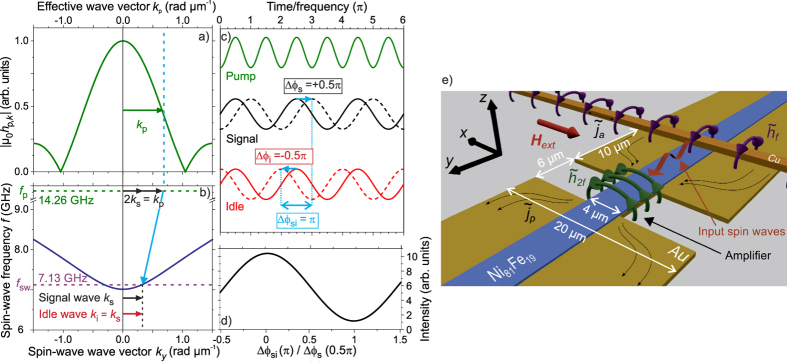
Working principle and concept of the phase-to-intensity conversion. (**a**) Due to the finite size of the amplifier, it provides a spectrum of effective wave vectors *k*_p_ (green line). (**b**) Microwave photons with *k*_p_ > 0 can split into co-propagating magnon pairs, leading to the formation of the signal and idler waves at one half of the pumping frequency. The solid dark blue line represents the spin-wave dispersion relation in the investigated Ni_81_Fe_19_ waveguide. (**c**) For a pumping field with fixed reference phase, schematically illustrated by the green line, the signal (black) and idler (red) waves are initially in-phase (solid lines). In this case, their interference is constructive. If the phase of the signal wave is shifted by *π*/2, the phase of the idler wave has to adjust its phase by −*π*/2 (dashed lines). Consequently, their interference becomes destructive. (**d**) Output intensity of the amplifier resulting from this interference mechanism. (**e**) Scheme of the experimentally investigated localized spin-wave amplifier together with its relevant dimensions. For details, see description in the main text.

The momentum of the microwave photons is negligibly small compared to the momentum of the spin waves. This generally leads to the creation of counter-propagating waves running in opposite direction to satisfy momentum, a process also referred to as adiabatic parallel pumping. However, if the microwave pumping field is spatially confined, this confinement leads to an effective-wave-number spectrum of the microwave photons. Figure 1(a) shows the Fourier spectrum of a 6 *μ*m long, rectangular field-distribution, which is a good approximation of the pumping field created within the actual amplifier used in the present experiment. As can be seen from the figure, the confinement leads to the existence of effective wave vectors *k*_p_ of the pumping field. As sketched in Fig. 1(b), where the spin-wave dispersion in the investigated Ni_81_Fe_19_ waveguide is shown, this enables the splitting of a microwave photon with *k*_p_ = 2*k*_s_ into a pair of signal and idler waves featuring *f*_s_ = *f*_i_ and propagating in the same direction. Here, it should be noted that the effective wave vector of the pumping field *k*_p_ = 2*k*_s_ does not imply that the pumping field consists of a propagating wave. The spatial dependence accounts for the fact that at each point in space the pumping field provides the necessary quasi-momentum to satisfy Eq. 1, i.e.,


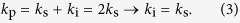


Thus, in this process of *non*-*adiabatic parametric amplification*^22^, pairs of co-propagating signal and idler waves can be created and their interference can be modified by changing the relative phase between the signal spin waves and the pumping field. This is due to the fact that the phase of the idler wave *ϕ*_i_ is linked to the phase of the signal wave *ϕ*_*s*_ = *k*_s_*y* + 2*πf*_s_*t* + *ϕ*_s,0_ and the phase of the pumping field *ϕ*_p_ = 2*k*_s_*y* + 4*πf*_s_*t* + *ϕ*_p,0_ via Eq. 2. Here, *ϕ*_p,0_ is the phase-offset of the pumping field, which is controlled externally by the pumping source. *ϕ*_s,0_ is the phase-offset of the signal wave. It is determined by the phase of the spin-wave excitation and the history of the spin wave propagation through the magnonic conduit and corresponds to the information carried by the wave-phase. For instance, if the signal wave is passed through a phase shifter, *ϕ*_s,0_ and, thus, its logic value, are changed. Defining the relative phase between the signal spin waves and the pumping field as Δ*ϕ*_sp_ = 2*ϕ*_s_ − *ϕ*_p_ = 2*ϕ*_s,0_ − *ϕ*_p,0_, the phase difference between the signal and the idler waves Δ*ϕ*_si_ = *ϕ*_s_ − *ϕ*_i_ is given by


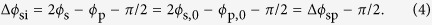


Thus, the phase difference between signal and idler waves is entirely determined by the phase-offset Δ*ϕ*_sp_. Hence, the phase of the idler wave is highly sensitive to a change in the relative phase between the signal wave and the pumping source. This constitutes the basic concept of the phase-to-intensity conversion discussed in this work.

This working principle is schematically illustrated in Fig. 1(c), where the time-evolution of the pumping field and the signal and idler waves are sketched for the two extreme cases (the individual amplitudes are arbitrary). The green solid line represents the temporal evolution of the pumping field, which acts as the reference phase. The solid black and red lines represent the phase-evolution of the signal and the idler wave, respectively, for Δ*ϕ*_sp_ = *π*/2 and, consequently, Δ*ϕ*_si_ = 0. For these phase relations, the created signal and idler waves interfere constructively. Hence, a large output of amplified waves is created. In this scenario, the amplifier works the most efficient and a large spin-wave amplification is observed. In contrast, if the phase of the signal wave is shifted by Δ*ϕ*_s_ = *π*/2 (black dashed line), the phase of the idler wave has to compensate this phase shift in order to fulfil Eq. 2, i.e., its phase is shifted by Δ*ϕ*_i_ = −*π*/2 (red dashed line). In this scenario, their phases differ by Δ*ϕ*_si_ = *π* (Δ*ϕ*_sp_ = 3/2*π*) and their interference becomes destructive. Due to this destructive interference process and since the pumping creates an equal amount of signal and idler waves, these waves cancel each other and there is no net output of amplified waves behind the amplifier. It should be noted that the non-adiabatic amplification process can still lead to the amplification of counter-propagating waves. The relative amount of co- to counterpropagating waves depends on the size of the pumping area in comparison to the spin-wave wavelength and the applied supercriticality. This results from the fact that the effective pumping field for the creation of the co-propagating waves consists of the pumping field components *h*_0_ + *h*_2*k*_, i.e., the field’s Fourier component *h*_0_ that allows for the amplification of counterpropagating waves as well as the component *h*_2*k*_, which allows for the creation of copropagating waves and which depends on the extents of the amplifier^22^,^23^. In contrast, the creation of counterpropagating waves only profits from the component *h*_0_ and, thus, requires a larger applied pumping field to exceed the threshold. Since in our experiment we are just above the threshold for the amplification of copropagating waves, the amount of counterpropagating waves appears negligible.

In this scenario and with amplitudes *A*_s_(*t*) of the signal wave and *A*_i_(*t*) of the idler wave, the time-averaged output intensity of the amplifier at a fixed readout position is given by


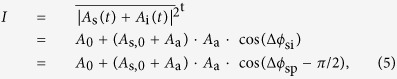


with the constant 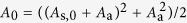. Here, *A*_s,0_ denotes the amplitude of the incoming signal wave and *A*_a_ the amplitude of the created signal as well as idler waves. The output of the amplifier according to this equation is shown in Fig. 1(d) assuming that the amount of signal and idler waves generated by the parallel pumping process is comparable to the strength of the incoming signal wave (*A*_a_ = 1.1 *A*_s,0_). By changing the phase shift Δ*ϕ*_sp_ and by the consequent change of Δ*ϕ*_si_, the output of the amplifier can be continuously tuned and, even for this rather small amount of amplified waves, a large modulation by a factor of about 10 can be achieved. If a notable amount of counterpropagating waves is created by the parametric amplification process, this leads to an additional offset in Eq. 5 since in this scenario, the amount of idler and signal waves created by the pumping does not coincide any more.

## Demonstration of the phase-to-intensity conversion

Based on these considerations, the investigated device, which is sketched in Fig. 1(e), consists of a Au micro-strip (regular width *w*_1_ = 20 *μ*m) carrying a microwave current 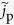. This current provides the dynamic Oersted field 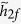 used for the parametric amplification. The current density is increased in a narrowed region of the strip, where the width is decreased to *w*_n_ = 4 *μ*m over a distance of *l*_n_ = 6 *μ*m. This increase in the current density results in a local enhancement of the generated pumping field 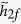 inside the Ni_81_Fe_19_ spin-wave waveguide, which is patterned on top and in the centre of the Au strip and which is transversely magnetized by a static external field of *μ*_0_*H*_ext_ = 63 mT. The peak power of the microwave current is chosen such that the parametric instability threshold is only just exceeded in the region of the enhanced microwave field. This leads to a strong spatial localization of the pumping process which provides the needed effective wave vector *k*_p_. Thus, this region is the actual local parametric amplifier^36^,^37^.

To excite a coherent spin wave in the Ni_81_Fe_19_ waveguide, which acts as the input signal wave with the information encoded in its phase, a microstructured Cu antenna is patterned on top of the magnonic waveguide at a distance of *l*_1_ = 13 *μ*m from the centre of the local parametric amplifier. A microwave current 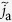 provides the Oersted field 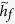 used for the direct spin-wave excitation at the antenna.

In the performed experiment, a Δ*t*_a_ = 50 ns long microwave pulse with a carrier frequency of *f*_s_ = 7.13 GHz provides the input spin-wave excitation at the antenna. The phase-offset *ϕ*_s,0_ of the signal spin wave is determined by the phase of this microwave pulse. In further experiments, which are discussed in the supplementary material (cf. Fig. S3), the wavelength *λ*_s_ of the excited spin wave at the applied field-frequency-combination was determined to be 13.5 *μ*m. The extent *l*_p_ of the localized pumping field is in the range of the spatial extent *l*_n_ = 6 *μ*m of the narrowing of the Au strip, since only in this region the threshold of parallel pumping is exceeded^37^. Hence, the conditions for the non-adiabatic parallel pumping process are fulfilled. After the signal wave has reached the amplifier, the pumping field 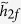 is provided by a second pulsed microwave current with a duration of Δ*t*_p_ = 50 ns flowing through the Au strip. Both microwave pulses are generated by the same microwave source. The pumping pulse with twice the frequency *f*_p_ = 2*f*_s_ = 14.26 GHz is created using a frequency doubler. Therefore, the phase difference Δ*ϕ*_sp_ = *ϕ*_s,0_ − *ϕ*_p,0_ between the pumping field and the signal wave and, consequently, the resulting phase difference Δ*ϕ*_si_, are stable and can be controlled by a phase shifter located in the microwave setup in the branch of the pumping pulse (cf. methods section).

**Figure 2:**
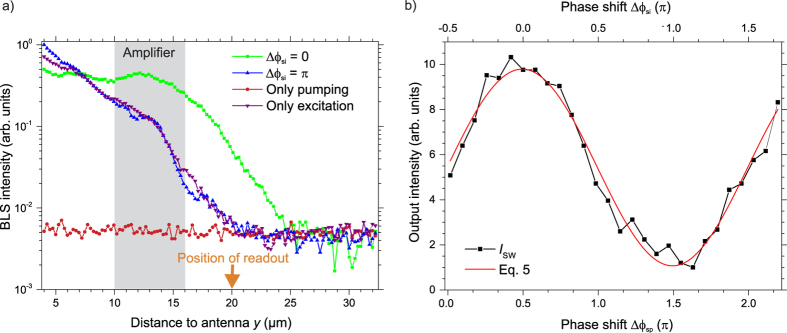
(**a**) BLS intensity (logarithmic scale) as a function of the distance to the micro-strip antenna. Green rectangles and blue upward triangles: Spin-wave intensity arising from the interplay between the parametric amplification and the spin-wave excitation. Between these two measurements, the phase of the pumping field *ϕ*_p,0_ was shifted. Red circles: Pumping only. Violet downward triangles: spin-wave excitation at the antenna only. The shading represents the spatial extent of the amplifier. (**b**) Intensity measured at a distance of 7 *μ*m (marked by the orange arrow in (**a**)) behind the centre of the amplifier as a function of the induced phase shift Δ*ϕ*_sp_ between the signal wave and the pumping field as well as of the resulting phase shift Δ*ϕ*_si_ between the signal and the idler waves. The red line represents a fit according to Eq. 5.

As a proof of concept of the phase-dependent amplification, Fig. 2(a) shows the BLS intensity (log-scale) measured in the centre of the waveguide as a function of the distance to the antenna in different scenarios. Here, the shaded area represents the geometric extent of the amplifier. The green squares and the blue upward triangles show the intensity arising from the interplay of the parallel pumping process and the externally excited signal spin wave. Between these two measurements, the phase of the pumping field was shifted by −*π*. As expected from Eq. 5, this has a striking influence on the amplification: In the first case, shown by the green squares, the phase between the pumping and the spin waves has been adjusted to Δ*ϕ*_sp_ = *π*/2, i.e., Δ*ϕ*_si_ = 0. Hence, the signal and the idler waves interfere constructively. Consequently, the BLS intensity grows within the amplifier as a result of the parametric amplification and a large output intensity is created. In contrast, if the phase of the pumping field is shifted by −*π* (blue upward triangles), this results in a destructive interference between signal and idler waves (Δ*ϕ*_sp_ = 1.5*π*, Δ*ϕ*_si_ = *π*, cf. Eq. 5). According to Eq. 5, the detected intensity is only due to the unamplified signal wave and no notable amplification should be observed. To confirm this, the violet downward triangles show the spin-wave intensity arising solely from the direct excitation by the antenna in the absence of parallel pumping. Indeed, the intensity of the spin waves leaving the local amplifier in the case of amplification with destructive interference coincides well with the intensity of the incoming, unamplified signal spin waves. The notable difference of the intensity in front of the amplifier by about ±35% is likely mediated due to the interference of the incoming signal spin waves with spin waves reflected during their propagation past the amplifier or with spin waves created by a weak amplification of counterpropagating waves. For completeness, the red circles show the intensity arising if only parallel pumping is applied, which leads to the amplification of thermal spin waves, a process known as parametric generation^36^,^38^,^39^. As can be seen from the figure, for the given pumping power and pulse duration, no rise of the BLS intensity within the amplifier due to parametric generation is noticeable. This is due to the fact that the applied microwave pulse is not long enough to amplify the thermal waves from their seed level above the detection limit of the BLS. Thus, the operated parametric amplifier does not generate a significant amount of noise.

This demonstrates the principal functionality to convert the phase of the spin waves into intensity information in a micron-sized spin-wave device. Distinct phase shifts of the signal spin wave are conveyed into an intensity variation after passing the amplifier by keeping the initial phase of the pumping field unchanged. The squares in Fig. 2(b) represent the detected spin-wave intensity as a function of the phase difference Δ*ϕ*_sp_ at a distance of 7 *μ*m behind the centre of the amplifier together with a fit according to Eq. 5. It can clearly be seen that the intensity of the signal wave can be continuously modulated by a factor of 10 by changing this phase difference. Hence, in this low-noise operating regime, the non-adiabatic parametric amplifier enables the convenient readout of the spin-wave phase in a magnonic circuit.

## Application within a magnonic network

**Figure 3:**
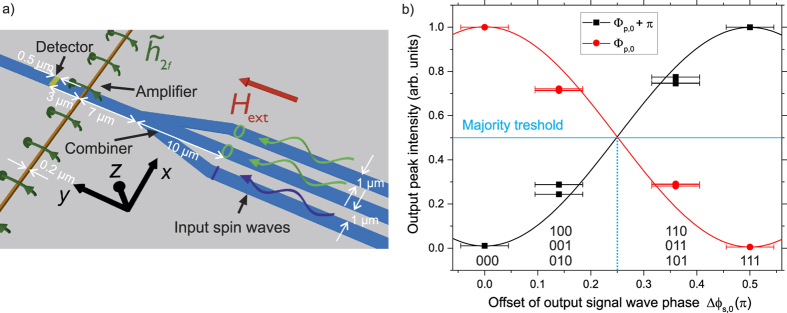
(**a**) Schematic of the simulated majority gate featuring a parametric amplifier to facilitate the readout of the phase information and relevant dimensions of the structure. The wavy arrows represent input spin waves with Δ*ϕ*_s,0_ = 0 (logic 0, green) and Δ*ϕ*_s,0_ = 0.5*π* (logic 1, blue), respectively. (**b**) Output spin-wave intensity as a function of the offset of the phase of the signal spin wave Δ*ϕ*_s,0_ in the output waveguide represented by red circles (reference phase *ϕ*_p,0_) and black squares (reference phase *ϕ*_p,0_ + *π*) (see Fig. S2 in the supplementary material for more detail). The solid lines represent Eq. 5.

Finally, we show the application of the phase-to-intensity conversion to an all-magnonic majority gate by means of micromagnetic simulations to give an instructive example on how this technique can be used in a magnonic network. We demonstrate how the conversion allows for a convenient and versatile performance of the majority function. A majority gate consists of an odd number of inputs and its output is given by the majority of the input values^10^,^40^. The use of waves allows to perform this within a single device, due to the fact that the majority information is intrinsic to the interference of an odd number of incident waves with well-defined phase relations. The simulations were performed using the micromagnetic simulation tool MuMax3^41^. The investigated majority gate incorporates a parametric amplifier^42^ and is based on the gate geometry shown in ref. 11, which is depicted in Fig. 3(a) (for details see supplementary material). The simulations have been performed in a structure based on the ferrimagnetic insulator yttrium iron garnet (YIG). Due to the very low spin-wave damping in this material, it allows for a spin-wave propagation in extended magnonic networks^43^,^44^,^45^,^46^,^47^. This way, the gate can be designed on length-scales which are easily accessible in a BLS experiment and easy to fabricate, whereas for an application to Ni_81_Fe_19_, a much smaller gate geometry is needed due to the shorter spin-wave decay length. In addition, the change of the geometry to longitudinally magnetized waveguides is motivated by the mode-selection mechanism presented in ref. 11, which profits from the larger splitting of the waveguide modes in this geometry.

For compliance with the phase-to-intensity conversion by parallel pumping, which is invariant to changes in the spin-wave phase by *π*, a logic 0 is encoded into an offset of the spin-wave phase of Δ*ϕ*_s,0_ = 0, whereas a logic 1 corresponds to an offset of Δ*ϕ*_s,0_ = 0.5*π*. Consequently, the interference of the three input waves in the output waveguide will lead to an output-phase of the signal wave in the output waveguide featuring Δ*ϕ*_s,0_ = 0 for the full majority of 0 (input values 000, i.e., logic 0 in each input), Δ*ϕ*_s,0_ = atan(0.5) ≈ 0.15*π* for the partial majority of logic 0 (e.g. 010), Δ*ϕ*_s,0_ = atan(2) ≈ 0.34*π* for the partial majority of logic 1 (e.g. 101) and Δ*ϕ*_s,0_ = 0.5*π* for a full majority of logic 1 (111). In combination with the non-adiabatic parametric amplifier, this will translate into 4 distinct intensity levels in the output.

Figure 3(b) shows the simulated output intensity of the gate arising from the phase-dependent amplification process for all possible input combinations for two different reference values of the pumping phase. The solid lines represent the expected output intensity according to Eq. 5. For the reference phase *ϕ*_p,0_, the pumping predominantly amplifies spin waves carrying no offset in their phase (logic 0) (red), whereas for *ϕ*_p,0_ + *π*, spin waves with an offset of Δ*ϕ*_s,0_ = 0.5*π* (logic 1) are preferably amplified (black). As can be seen from the figure, this change of the reference phase allows to select, which full majority - 000 or 111 - is leading to a maximum intensity behind the amplifier. In addition, it allows to interchange the intensity levels of the partial majorities (e.g. 010 and 101). By defining the majority by an intensity larger than the intensity threshold depicted in Fig. 3(b), which is situated at one half of the intensity of the full majority, the majority operation is preserved. Hence, a parametric amplifier connected to such a majority gate translates the majority of the input phases into an amplified spin-wave intensity at the output. Hereby, the preferred phase can be selected by adjusting the reference phase of the pumping. In addition, from the intensity, it can be concluded whether the majority was full or partial. This is one instructive example on how the use of a non-adiabatic parametric amplifier can be implemented into a magnonic circuit to achieve an enhanced functionality and to facilitate the readout of the phase-information.

## Conclusions

To conclude, we have experimentally proven the possibility to convey the phase of a magnonic spin current into a spin-wave intensity directly within the spin-wave system on a microscopic scale. This is achieved by the phase-dependent amplification of coherent spin waves propagating in a microstructured Py waveguide by means of localized, non-adiabatic parallel pumping. We find a strong phase-dependent modulation of the output intensity by a factor of 10. This ensures a high reliability of the readout process and increases the sensitivity for the detection of small spin-wave intensities with proper phase. For the purpose of information processing, this can be used as a scalable approach to readout the phase of spin waves in devices with length scales and materials that meet the requirements for applications. With this, the spin-wave intensity can be kept at a minimum during signal processing to avoid nonlinear magnon-magnon interaction and to reduce the energy costs while, for the readout, spin waves are amplified to a detectable limit. By means of micromagnetic simulations, we have demonstrated how this allows for the versatile operation of a magnon-based majority gate. These results demonstrate the active role a parametric amplifier can play in a magnonic circuit. The phase-to-intensity conversion paves the way for the interconnection of spin-wave logic circuits with electronic circuits which relies on the convenient DC detection of the wave-phase decoded into the amplitude of the detected signal.

## Methods

### Sample fabrication

The pumping strip-line (regular width *w*_1_ = 20 *μ*m) with the constriction (width *w*_n_ = 4 *μ*m, length *l*_n_ = 6 *μ*m) is defined by electron-beam lithography (EBL) in a lift-off process from Cr/Au (10 nm/200 nm). The strip is u-shaped and connected to larger pads which allow for the contact with high-frequency probes. Subsequently, a 250 nm thick Hydrosilesquioxane layer is spin-coated onto the sample and patterned by EBL into a rectangular pad to smoothen the roughness of the pumping strip-line and to electrically insulate it from the structures to be patterned on top. A large EBL dose is used to cross-link the Hydrosilesquioxane pad. Subsequently, the 40 nm thick Ni_81_Fe_19_ layer with a MgO (5 nm) capping layer is sputter-deposited on top of a Ru/MgO (7.5 nm/5 nm) buffer layer and patterned into the *w*_w_ = 4 *μ*m wide and *L*_w_ = 75 *μ*m long waveguide by EBL and ion-beam etching. In a final step, the micro-strip antenna is fabricated. For this, a shunted coplanar waveguide is patterned by EBL and a lift-off process from Ti/Cu (10 nm/500 nm), where a *w*_a_ = 2 *μ*m wide shortcut constitutes the actual antenna. This coplanar waveguide is also connected to larger pads for contacting to high frequency probes.

### Experimental techniques

**Figure 4:**
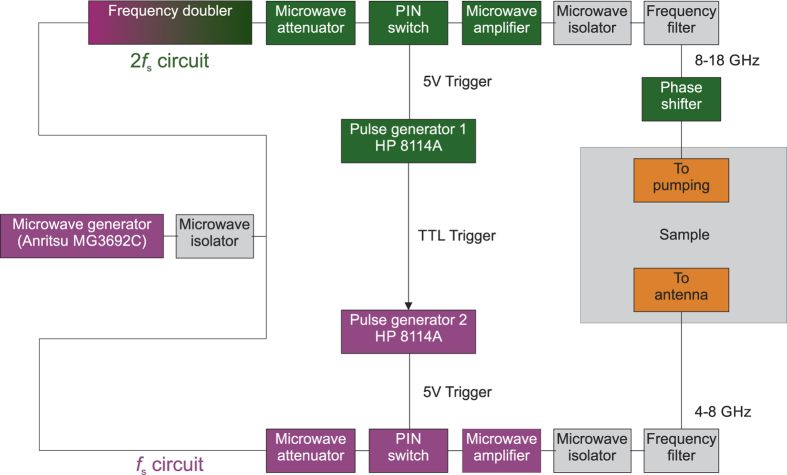
Sketch of the used microwave setup.

The sample is connected to the microwave setup as schematically shown in Fig. 4. A microwave source is used to create a CW microwave signal at the carrier frequency *f*_s_. After passing a microwave isolator, the signal is split and one half of the signal is frequency-doubled. Subsequently, both signals are chopped into pulses by PIN-switches which are triggered by synchronized pulse generators. In front of the PIN switches, attenuators are installed to allow for an adjustment of the microwave peak powers. After pulsing, the signals are amplified by *P*_amp_ = +30 dB. Consequently, each of the microwave pulses passes another microwave isolator and is frequency filtered before being passed to the high-frequency probes, which are in contact with the sample. The peak-microwave power of the spin-wave excitation in the presented experiment was *P*_a_ = 5 dBm, the peak pumping power *P*_p_ = 22 dBm. A phase-shifter is installed in front of the sample in the 2*f*_s_ branch, which allows for an external control of the phase difference Δ*ϕ*_sp_.

The spin-wave dynamics which are excited by this microwave setup are analysed by means of Brillouin light scattering microscopy. A solid-state laser provides the reference light at a wavelength of 532 nm, which is guided onto the sample by a microscope objective and focused down to a spot size of about 430 nm. The scattered light is re-collected by the objective and guided into a 3 + 3-pass JRS Tandem Fabry Pérot interferometer^48^, where it is analysed with respect to frequency and intensity. The obtained BLS intensity at a given frequency is proportional to the number of inelastic scattering events of the light with magnons in the probing spot, which is directly proportional to the local spin-wave intensity.

### Micromagnetic simulations

The micromagnetic simulations are carried out using the GPU-based micromagnetic code MuMax3^41^. An area of 40 × 5 *μ*m^2^ is simulated in the *x*–*y*-plane with a discretization into 4096 × 512 cells. Perpendicular to the *x*–*y*-plane (*z*-direction), one cell of a fixed thickness of 100 nm is assumed, representing the homogeneous magnetization across the thickness of the YIG film. Globally, the material parameters of thin YIG have been used^43^: *M*_s_ = 140 kAm^−1^, Gilbert damping *α* = 0.0005, and an exchange constant of *A*_ex_ = 3.5 pJm^−1^. Towards the borders of the simulated area in the *y*-direction, the damping is enlarged to dampen out spin waves reaching this region to avoid their reflection. For more information, see the Supplementary Material.

## Additional Information

**How to cite this article**: Brächer, T. *et al*. Phase-to-intensity conversion of magnonic spin currents and application to the design of a majority gate. *Sci. Rep.* **6**, 38235; doi: 10.1038/srep38235 (2016).

**Publisher’s note:** Springer Nature remains neutral with regard to jurisdictional claims in published maps and institutional affiliations.

## Supplementary Material

Supplementary Material
