# Supplementary material for: Phase-to-intensity conversion of magnonic spin currents and application to the design of a majority gate
Source: Sci Rep. 2016 Dec 1;6:38235. doi: 10.1038/srep38235 (PMC5131322; doi:10.1038/srep38235)
Supplement: Supplementary Material [file srep38235-s1.pdf]

Supplementary material to manuscript “Phase-to-intensity conversion of magnonic spin currents and application to the design of a majority gate”

T. Brächer[1,2,3,*,+], F. Heussner[3,+], P. Pirro[3,+], T. Meyer[3], T. Fischer[2,3], M. Geilen[3], B. Heinz[3], B. Lägel[3], A. A. Serga[3], and B. Hillebrands[3]

[1]Univ. Grenoble Alpes, CNRS, CEA, INAC-SPINTEC, 17, rue des Martyrs 38054, Grenoble, France
[2]Graduate School Materials Science in Mainz, Gottlieb-Daimler-Strasse 47, D-67663 Kaiserslautern, Germany
[3]Fachbereich Physik and Forschungszentrum OPTIMAS, Technische Universität Kaiserslautern, D-67663 Kaiserslautern, Germany
[*]thomas.braecher@cea.fr
[+]This authors contributed equally to this work

ABSTRACT

In this supplementary material we provide information about the micromagnetic simulation of the phase-to-intensity conversion applied to an all-magnonic majority gate performed by MuMax3. In addition, it describes the procedure used to determine the spin-wave wave vector in the experiment.

Micromagnetic simulation of the phase-to-intensity conversion applied to an all-magnonic majority gate

As sketched in Fig. 3 (a) in the manuscript, the studied gate consists of three spin-wave inputs where the logic information is encoded in phase-offsets of the incident spin waves[1,2]. In the output waveguide, the majority function is directly realized via the interference of the input waves, i.e., the majority of the phase of the input waves determines the phase of the output signal wave. The corresponding truth table is shown in Tab. S1. As can be seen from the table, if the majority of the inputs corresponds to a logic 0, the output is 0, whereas it is 1 if the majority of the input values is equal to a logic 1.

The simulations of the majority gate have been performed using the micromagnetic simulation tool MuMax3[3]. An area of $40 \times 5\,\mu\mathrm{m}^2$ is simulated in the $x-y$-plane with a discretization into 4096×512 cells. Perpendicular to the $x-y$-plane (z-direction), one cell of a fixed thickness of 100 nm is assumed, representing the homogeneous magnetization across the thickness of the magnetic film.

To model the gate, the material parameters of the ferrimagnetic insulator yttrium iron garnet (YIG), a material which excels due to its extra-ordinarily low spin-wave damping, have been used[4]: Saturation magnetization $M_\mathrm{s} = 140\,\mathrm{kA\,m^{-1}}$, Gilbert damping $\alpha = 0.0005$ and an exchange constant of $A_\mathrm{ex} = 3.5\,\mathrm{pJ\,m^{-1}}$. In contrast to $Ni_{81}Fe_{19}$, YIG allows for a long-range spin-wave propagation and enables the performance of complex interference experiments since the spin-wave decay length in YIG greatly exceeds the spin-wave wavelength. Recently, the experimental realization of spin-wave excitation and propagation in microstructures made from thin YIG films was reported[4–8], promising the successful application of magnonic networks made from YIG on the microscopic scale.

The gate consists of three $1\,\mu$m wide, $15\,\mu$m long input waveguides which are merged into a $25\,\mu$m long, $1\,\mu$m wide output waveguide. An external field of $\mu_0 H_\mathrm{ext} = 50\,\mathrm{mT}$ is applied along the long axes of the waveguides. In each input, propagating, coherent spin waves are continuously excited at a frequency of $f_\mathrm{s} = 2.8\,\mathrm{GHz}$ with well-defined initial phases. The excitation in the input waveguides is modelled by means of coplanar waveguides (CPW, not shown in Fig. 3 (a) in the manuscript). The simulated CPWs feature a width of $w_\mathrm{CPW} = 800\,\mathrm{nm}$ and a thickness of $h_\mathrm{CPW} = 250\,\mathrm{nm}$ of the current carrying wires with a centre-to-centre distance of $d_\mathrm{CPW} = 1\,\mu\mathrm{m}$. They are situated at a distance of $7\,\mu$m from the beginning of the combiner region in which the three inputs are merged. The spin-wave dispersion relations of the lowest waveguide modes $n = 1$ (fundamental mode) - $n = 3$ in the $1\,\mu$m wide inputs and the output are shown in Fig. S1, together with the spin-wave wave vector $k_{||} \approx 9.43\,\mathrm{rad}\mu\mathrm{m}^{-1}$ excited by the CPWs extracted from the micromagnetic simulations. As can be seen, the dispersion of the mode $n = 2$ lies well above this frequency, which ensures that only the mode $n = 1$ can propagate in the waveguides, in accordance with the mode-selectivity mechanism employed in Ref.[1].

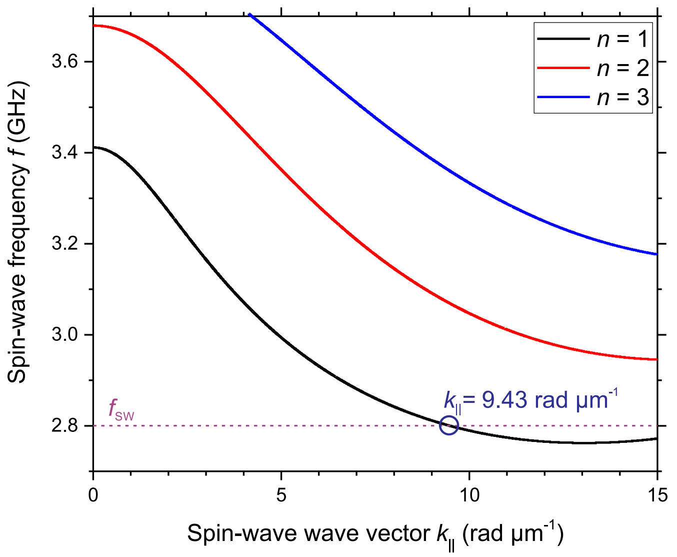

Figure S1. Spin-wave dispersion relation of the three lowest waveguide modes $n=1$ (fundamental mode) - $n=3$. The dispersion has been calculated following Ref.[14], using the material parameters stated in the text and an effective waveguide width $w_{\text{eff}} = 1.13\,\mu$m, which accounts for dipolar pinning and the exchange interaction[15].

The readout is mimicked by integrating the spin-wave intensity $10\,\mu$m behind the combiner over a $1\,\mu$m wide and 500 nm long (y-direction) rectangle. To obtain the output at $f_\text{s} = 2.8$ GHz, the acquired temporal dynamics of the out-of-plane magnetization component m_z have been Fourier transformed, filtered in frequency, and, by inverse FFT, these filtered data were converted into time traces. This procedure is not needed to observe the phase-to-intensity conversion discussed in the manuscript. It is helpful, however, in order to acquire the clean temporal temporal dynamics at 2.8 GHz.

To apply the phase-to-intensity conversion to a spin-wave based majority gate, it has to be kept in mind that the phase-dependent amplification of the parametric amplifier is invariant to a change in the signal spin-wave phase by π. In the majority gates presented in Refs.[1,2], an offset of the spin-wave phase of $\Delta\phi_{\text{s},0} = 0$ was associated with a logic 0 and an offset of $\Delta\phi_{\text{s},0} = \pi$ was associated with a logic 1. Hence, for the compliance with the phase-to-intensity conversion by parallel pumping, the encoding and its interpretation have to be adapted. If a logic 1 is encoded in a phase-offset of $\Delta\phi_{\text{s},0} = 0.5\pi$ and with equal amplitudes A of all three waves entering the output waveguide, their interference results in:

$$\begin{aligned}
A_{000} &= 3A\sin(\phi(y,t)) \\
A_{111} &= 3A\sin(\phi(y,t)+0.5\pi) = 3A\cos(\phi(y,t)) \\
A_{100} &= A_{010} = A_{001} = 2A\sin(\phi(y,t)) + A\cos(\phi(y,t)) \\
&= \sqrt{5}A\sin(\phi(y,t)+\text{atan}(0.5)) \\
A_{110} &= A_{101} = A_{011} = 2A\cos(\phi(y,t)) + A\sin(\phi(y,t)) \\
&= \sqrt{5}A\sin(\phi(y,t)+\text{atan}(2)).
\end{aligned} \tag{1}$$

With the resulting phase offsets $\Delta\phi_\text{s}$, the truth table Tab. S1 can be reproduced if all phase-offsets in the output waveguide which are above and below 0.25π are treated as logic 1 and 0, respectively.

To demonstrate this working principle of the majority function, the output of the majority gate in the absence of parallel pumping is shown in Fig. S2 (a). The figure shows the time-resolved amplitude of the dynamic out-of-plane magnetization component m_z at $f_\text{s} = 2.8$ GHz in the detector-region during one oscillation period for all possible input combinations of the majority gate. As can be seen from the figure, 000 (green curve) and 111 (blue curve) are shifted in phase by $\pi/2$. All other input combinations feature phase-offsets at intermediate values ($(0.14\pm0.02)\pi$ and $(0.36\pm0.02)\pi$, respectively) which are in excellent agreement with the expectations according to Eqs. 1. Thus, by treating the phase-offset as a continuous variable and by discriminating a logic 0 and 1 around a threshold phase-offset of 0.25π, the majority operation is maintained.

To model the phase-to-intensity conversion by non-adiabatic parallel pumping, a 200 nm wide, 250 nm thick micro-strip-antenna has been simulated at a distance of $7\,\mu$m behind the combiner region. Since the gate - besides the combiner - consists of longitudinally magnetized spin-wave waveguides, such a micro-strip-antenna can be used as a localized source of a microwave Oersted field properly oriented for parallel pumping[9]. The width of this antenna is well below the wavelength

Table S1. Truth table of the simulated majority gate, stating the output values of the gate for all possible input combinations. The values in brackets represent the resulting phase-offset $\Delta\phi_s$ of the signal spin waves in the output waveguide extracted from a simulation without parallel pumping. A logic 0 in the input is encoded into a phase-offset of 0 and a logic 1 into a phase-offset of 0.5π. All phase-offsets in the output larger than 0.25π are identified with a logic 1.

Input 1	Input 2	Input 3	Output
0	0	0	0 (0.0π)
1	0	0	0 $(\text{atan}(0.5) \approx 0.15\pi)$
0	1	0	0 (0.15π)
0	0	1	0 (0.15π)
1	1	0	1 $(\text{atan}(2) \approx 0.35\pi)$
1	0	1	1 (0.35π)
0	1	1	1 (0.35π)
1	1	1	1 (0.5π)

$\lambda_s \approx 666$ nm of the signal waves excited in the inputs. Thus, the parametric amplification is non-adiabatic. A microwave pulse with an amplitude of $\mu_0\tilde{h}_{2f} = 62$mT, a duration of $\Delta t_p = 30$ ns and with a carrier frequency of $f_p = 2f_s = 5.6$ GHz is modelled, starting at $t = 180$ ns after the spin-wave excitation. This starting time ensures that the spin-wave amplitude is stationary in the output waveguide before the pumping is applied.

Figure S2 (b) shows the resulting time-resolved spin-wave intensity at $f_s = 2.8$ GHz integrated over the detector region for all possible input combinations. The intensity has been normalized to the maximum intensity reached for optimum amplification. The maxima of these time-traces are shown in Fig. 3 (b) in the manuscript. As expected, the pumping leads to a strongly phase-dependent amplification of the signal spin waves, where four distinct maximum intensity levels can be distinguished depending on the input-combination of the gate. The levels larger than 0.5 correspond to the full and partial majority of logic 0, whereas the levels below 0.5 correspond to the partial and full majority of logic 1, respectively. Thus, by setting the threshold to distinguish between majority 0 and 1 at half of the intensity of the full majority of 000, the majority function is translated into a spin-wave intensity. Moreover, by a readout of the absolute value of the intensity, it is possible to distinguish whether the majority was full (i.e., 000) or only partial (i.e., 010), since the overall intensity is proportional to the number of inputs with proper phase with respect to the pumping. In the inset of Fig. S2 (b), the phase of the pumping has been shifted by π, so

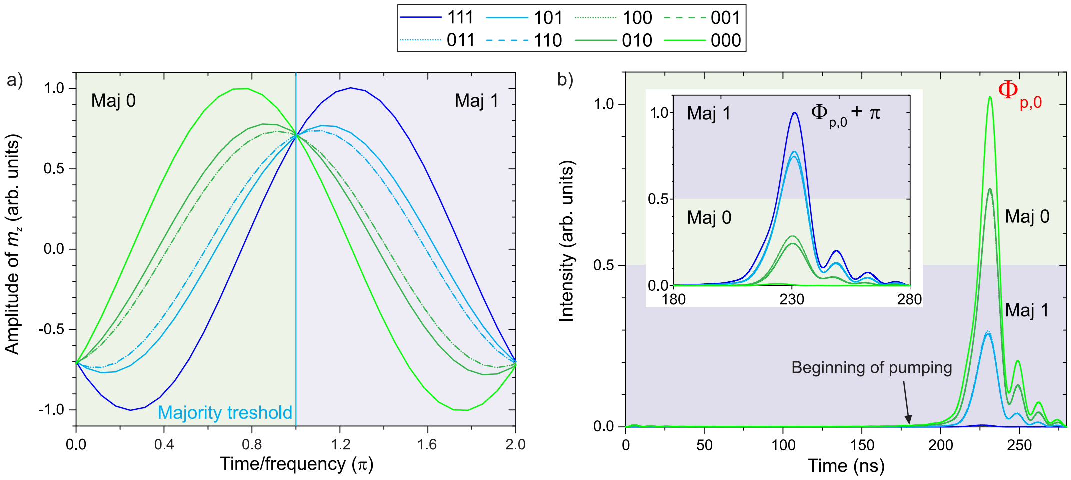

Figure S2. a) Time-dependence of m_z at $f_s = 2.8$ GHz at the detector for all input combinations of the gate in the absence of parallel pumping ($t \approx 180$ ns). A phase-offset above 0.25π is identified with majority 1. b) Time-dependent intensity at $f_s = 2.8$ GHz in the detector region in the presence of parallel pumping. The inset demonstrates the effect of a shift of the pumping phase by π.

that spin waves with a phase of 0.5π are preferably amplified. Consequently, the intensities become inverted and the largest intensity corresponds to a full majority of logic 1 (111). The error bars in Fig. 3 (b) in the manuscript contain the inaccuracy of the pumping phase $\phi_{p,0}$ as well as the error in $\Delta\phi_{s,0}$ visible in Fig. S2 (a) due to small differences in phase and amplitude between the central and the outer inputs (cf., e.g., 010 and 100). Within this error, the analytical description is in reasonable agreement with the simulated values of the output intensity (cf. Fig. 3 (b) in the manuscript). This demonstrates the ability to translate the majority information into a spin-wave intensity by parallel pumping.

Determination of spin-wave wavelength

Since the spin-wave wavelength of $\lambda = 13.5\,\mu$m at the external bias field $\mu_0 H_{ext} = 63\,$mT used in the experiment exceeds the exponential spin-wave amplitude decay length of about $3\,\mu$m significantly, the wavelength has been determined by a two step process: First the spin-wave wavelengths at smaller values of the externally applied bias field have been determined. These values have been used to fit the spin-wave dispersion relation. Consequently, the wavelength at $\mu_0 H_{ext} = 63\,$mT has been extracted from this dispersion relation.

Two different methods have been used to determine the spin-wave wavelength: The wavelengths were either extracted from the stationary intensity pattern arising from the interference of the spin waves with frequency-modulated light provided by an electro-optical modulator (i.e., the working principle of phase-resolved BLS[10,11]) or from the interference of two counter-propagating spin waves[12]. In the latter case, the spin waves running towards the antenna have been created by an *adiabatic* parallel pumping process, which takes place if the spin-wave wavelength is smaller than the spatial size of the amplifier[13]. Figure S3 (a) shows the interference patterns obtained from these two methods at an external bias field of $\mu_0 H_{ext} = 50\,$mT together with fits modelling the interference and the exponential decay. Both fits yield a spin-wave wavelength of about $5\,\mu$m (and an amplitude decay length of about $2.8\,\mu$m). Figure S3 (b) shows the experimentally determined wavelengths together with a fit of the dispersion relation resulting in a saturation magnetization $M_s = 810\,\mathrm{kAm}^{-1}$, thickness $d = 37\,$nm, effective width $w_{eff} = 3.6\,\mu$m, demagnetization field $\mu_0 H_{demag} = 6\,$mT and exchange constant $A_{ex} = 13\,\mathrm{pJm}^{-1}$. From this curve the wavelength at $\mu_0 H_{ext} 63\,$mT, i.e., $\mu_0 H_{eff} = 57\,$mT is determined to $\lambda = 13.5\,\mu$m ($k_{||} = 0.47\,\mathrm{rad}\mu\mathrm{m}^{-1}$). It should be noted that these measurements also proof that the parametric amplifier can be operated in the adiabatic regime[13] by a change of the applied bias field or the pumping frequency. In this regime of parametric amplification, the effective wave vector provided by the localization is not large enough to allow for the creation of co-propagating waves. Consequently, the output of the amplifier is independent of the relative phase between the signal spin waves and the pumping field.

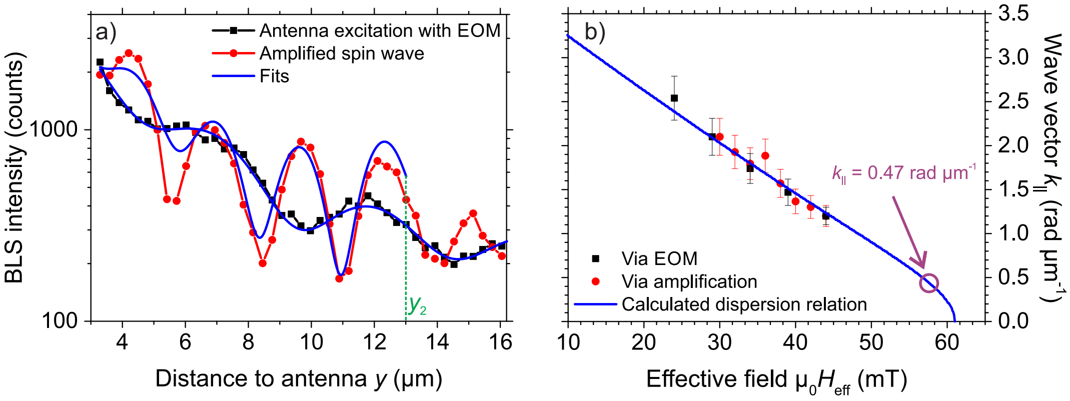

Figure S3. a) Exemplary stationary intensity patterns arising from the interference of the signal spin waves with the EOM (black) and the counter-propagating idler waves (red). y_2 denotes the point from which on the counter-propagating waves have been fitted and corresponds to the centre of the amplifier). b) Extracted values of the spin-wave wave vector together with the analytical spin-wave dispersion relation and the extrapolated wave vector at $\mu_0 H_{ext} = 63\,$mT, i.e., $\mu_0 H_{eff} = 57\,$mT.

References

1. Klingler, S. et al., Design of a spin-wave majority gate employing mode selection, *Appl. Phys. Lett.* **105**, 152410 (2014).

2. Klingler, S. et al., Spin-wave logic devices based on isotropic forward volume magnetostatic waves, *Appl. Phys. Lett.* **106**, 212406 (2015).

3. Vansteenkiste, A. et al., The design and verification of mumax3, *AIP Advances* **4**, 107133 (2014).

4. Pirro, P. et al., Spin-wave excitation and propagation in microstructured waveguides of yttrium iron garnet/Pt bilayers, *Appl. Phys. Lett.* **104**, 012402 (2014).

5. Hamadeh, A. et al., Full control of the spin-wave damping in a magnetic insulator using spin-orbit torque, *Phys. Rev. Lett.* **113**, 197203 (2014).

6. Hahn, C. et al., Measurement of the intrinsic damping constant in individual nanodisks of $Y_3Fe_5O_{12}$ and $Y_3Fe_5O_{12}$—Pt, *Appl. Phys. Lett.* **104**, 152410 (2014).

7. Jungfleisch, M. B. et al., Spin waves in micro-structured yttrium iron garnet nanometer-thick films, *J. Appl. Phys.* **117**, 17D128 (2015).

8. Yu, H. et al., Magnetic thin-film insulator with ultra-low spin wave damping for coherent nanomagnonics, *Scientific Reports* **4**, 6848 (2014).

9. Brächer, T., Pirro, P., Serga, A. A., and Hillebrands, B., Localized parametric generation of spin waves in a longitudinally magnetized $Ni_{81}Fe_{19}$ waveguide, *Appl. Phys. Lett.* **103**, 142415 (2013).

10. Fohr, F. et al., Phase sensitive Brillouin scattering measurements with a novel magneto-optic modulator, *Rev. Sci. Instrum.* **80**, 043903 (2009).

11. Vogt, K. et al., All-optical detection of phase fronts of propagating spin waves in a $Ni_{81}Fe_{19}$ microstripe, *Appl. Phys. Lett.* **95**, 182508 (2009).

12. Pirro, P. et al., Interference of coherent spin waves in micron-sized ferromagnetic waveguides, *Phys. Status Solidi B* **248**, 2404 (2011).

13. Melkov, G. A. et al., Parametric interaction of magnetostatic waves with a nonstationary local pump, *JETP* **89**, 1189 (1999).

14. Kalinikos, B. A. and Slavin, A. N., Theory of dipole-exchange spin wave spectrum for ferromagnetic films with mixed exchange boundary conditions, *J. Phys. C: Solid State* **19**, 7013 (1986).

15. Guslienko, K. Y., Demokritov, S. O., Hillebrands, B., and Slavin, A., Effective dipolar boundary conditions for dynamic magnetization in thin magnetic stripes, *Phys. Rev. B* **66**, 132402 (2002).
